# Screening of nontoxic dyes for improved visualization and success rate in mouse embryo transfer

**DOI:** 10.1186/s42826-025-00261-7

**Published:** 2025-12-01

**Authors:** Imai Hiroyuki, Matsuya Sumito, Uemura Tatsumi, Fujino Kaoru, Kawabe Toshiaki, Kano Kiyoshi, Kusakabe Ken Takeshi

**Affiliations:** 1 Laboratory of Veterinary Anatomy, Joint Faculty of Veterinary Medicine, Yamaguchi University, 1677-1 Yoshida, Yamaguchi, 753-8515 Japan; 2 Laboratory of Veterinary Anatomy, Joint Faculty of Veterinary Medicine, Kagoshima University, 1-21-24 Korimoto, Kagoshima, 890-0065 Japan; 3 ARK Resource Co., Ltd, 456 Osozu, Misato-Machi, Shimomashiki-gun, Kumamoto, 861-4401 Japan; 4 Laboratory of Veterinary Developmental Biology, Joint Faculty of Veterinary Medicine, Yamaguchi University, 1677-1, Yoshida, Yamaguchi, 753-8515 Japan

**Keywords:** Dye, Embryo transfer, Toxicity, Visibility

## Abstract

**Background:**

Advancements in genome editing technology and the globalization of life science research have highlighted the need for refined techniques in individualizing fertilized mouse eggs. Traditional embryo transfer surgery, which demands precise and objective success assessment, presents challenges for technical mastery and animal welfare. To address these issues, we developed a novel culture medium incorporating a dye library that enhances the visibility of ultrafine capillaries.

**Results:**

We screened 32 compounds based on their color properties and excluded those with clear cytotoxicity through colony formation inhibition tests. Five compounds demonstrated developmental outcomes comparable to those of the control group during embryo culture. Gene expression analysis revealed high correlation with control embryos, indicating no adverse developmental impact. No maternal toxicity was observed during and after embryo transfer using the dyed medium, which also produced normal offspring. A randomized survey confirmed improved visibility compared to conventional methods.

**Conclusion:**

This study successfully identified nontoxic dyes that enhance the visibility of embryo transfer culture media, potentially improving precision and animal welfare in embryological research. Based on our findings, adding Brilliant Blue FCF or Fast Green FCF to the medium is recommended.

**Supplementary information:**

The online version contains supplementary material available at 10.1186/s42826-025-00261-7.

## Background

Embryo transfer is vital in life science research for individualizing experimental animals. However, as researchers age, they face declining vision, impacting precision experiments such as embryo transfer. Particularly, the prevalence of presbyopia rises rapidly in individuals aged 40 and above [1], indicating that more than half of the full-time researchers in Japanese universities are susceptible to this risk [2]. Despite these societal factors, there has been little improvement in the techniques for embryo transfer techniques that rely on visual cues. Traditional methods such as manipulating a capillary with air bubbles [3], can introduce complications [4]. Although sporadic reports have emerged on non-surgical methods for embryo transfer [5, 6], they require specialized chips and catheter techniques and have not achieved widespread adoption due to comparable efficiency with conventional methods. To address the critical point of embryo transfer, we focused on improvements of embryo injection step.

To enhance visibility during embryo transfer, we explored food additive dyes. In vivo genome editing methods, such as GONAD and i-GONAD, initially utilized 0.05% trypan blue [7, 8]; however, recent studies switched to fast green (0.04–0.05%, final concentration) [9]. While these reports have shown successful births, they primarily focus on the success or failure of genome editing, without discussing the safety or effects of these dyes on embryos.

Therefore, in this study, we explored food additive dyes to enhance visibility without affecting embryonic development. We conducted investigations into the safety of these dyes to ensure their suitability for this purpose.

## Methods

### Reagents

The dye reagents and their concentrations used in this study are listed in Table 1. Stocks were prepared using DPBS (Dulbecco’s Phosphate Buffered Saline, Fujifilm Wako, Osaka, Japan). Quinizarin Blue (QB) and Quinizarin Green SS (QG) were dissolved in DMSO (Dimethyl Sulfoxide Hybri-Max, Sigma-Aldrich, MO, USA). For color analysis, the dyes were added to M16 medium (Sigma-Aldrich) and quantification was performed by dividing the images into red, green, and blue channels using ImageJ software (version 1.53, NIH, MD, USA).

**Table 1:** The dye used in this study

Abbreviations	Reagent	Company	Catalog Number	Final concentration (%)
ABI	Acid Black I	Tokyo Chemical Industry	A0586	0.02
ABM	Acid Brown M	Tokyo Chemical Industry	E0228	0.02
ACGF	Alizarin Cyanin Green F	Tokyo Chemical Industry	A0610	0.02
AG3	Acid Green 3	Tokyo Chemical Industry	G0176	0.02
AR	Acid Red 52	Tokyo Chemical Industry	A0600	0.01
BB	Brilliant Blue FCF	Fujifilm Wako	2712842	0.01
BBr	Bismarck Brown Y	Merck	861111	0.01
BCB	Brilliant Cresyl Blue	Cosmo Bio	15015	0.02
BPB	Bromophenol Blue	Fujifilm Wako	2102911	0.02
CSB	Chicago Sly Blue 6B	Santa Cruz	SC361146	0.01
EB	Evans Blue	Nacalai Tesque	915874	0.005
EM	Eumelanin	Fujifilm Wako	30231862	0.02
FG	Fast Green FCF	Fujifilm Wako	6100031	0.01
GB	Gardenia Blue	Fujifilm Wako	7303652	0.02
IC	Indigo Carmine	Fujifilm Wako	09000082	0.01
ICG	Indocyanine Green	Fujifilm Wako	155020	0.02
JG	Janus Green	Fujifilm Wako	10600011	0.02
LGY	Light Green SY Yellowish	Tokyo Chemical Industry	L0141	0.08
MB	Methylene Blue	Fujifilm Wako	13306962	0.02
MG	Methyl Green	Fujifilm Wako	13413901	0.02
NB	Nile Blue A	Merck	N5632	0.01
NC	New Coccine	Fujifilm Wako	14706542	0.02
NGB	Naphtol Green B	Fujifilm Wako	14000312	0.02
PB	Patent Blue	Fujifilm Wako	16111312	0.00125
Ph	Phloxine B	Fujifilm Wako	16602072	0.01
RB	Rose Bengal	Fujifilm Wako	18400272	0.01
SCC	Sodium Copper-chlorophylin	Fujifilm Wako	19801832	0.02
SY	Sunset Yellow FCF	Fujifilm Wako	19111072	0.01
T	Tartrazine	Fujifilm Wako	20400102	0.01
TB	Trypan Blue	Dojindo	34507421	0.005
QB	Quinizarin Blue	Tokyo Chemical Industry	Q0022	0.02
QG	Quinizarin Green SS	Tokyo Chemical Industry	Q0021	0.02

### Cell culture and toxicity

Cell toxicity tests followed the International Standardization Organization “Biological Evaluation of Medical Devices” standard [10]. Colony formation inhibition assays with V79 cells and comet assays were conducted [11, 12]. V79 cells were cultured in EMEM (Fujifilm Wako) supplemented with 10% FBS (Fetal bovine serum, Thermo Fisher Scientific, MA, USA). Approximately 100 cells/well were seeded in assay plates (TPP, BM Equipment, Tokyo, Japan) containing the test substance and cultured for one week. After fixation with methanol (Fujifilm Wako), colonies were stained with 5% Giemsa stain solution (Fujifilm Wako) in DPBS and counted. Relative colony numbers were calculated with the control condition set to 1. For the comet assay, cell suspensions cultured with the test substance were mixed with 0.5% low-melting point agarose (Agarose XP: Nippon Gene, Tokyo, Japan) in DPBS at a 1:10 ratio and spread onto glass slides. Following lysis using a buffer composed of 2.5 M NaCl (Fujifilm Wako), 1 mM EDTA (Ethylenediaminetetraacetic acid; Fujifilm Wako), and 1% N-Lauroylsarcosine (Fujifilm Wako) (pH10), supplemented with 1% Triton-X (Sigma-Aldrich) and 10% DMSO (Fujifilm Wako), electrophoresis was conducted at 25 V for 30 minutes at 4 °C in an alkaline solution (300 mM NaOH (NacalaiTesque, Kyoto, Japan) and 1 mM EDTA, pH > 13). The slides were then neutralized with Tris-HCl (Fujifilm Wako) and stained with GelGreen (Biotium, CA, USA) in DPBS.

### Animal experiment and embryo culture

Mouse embryos at the 2-cell stage were collected from BDF1 or ICR mice (Japan SLC, Hamamatsu, Japan) 1.5 days post-coitum (d.p.c.) and cultured in M16 medium with added dye solutions (Table 1) in a CO_2_ incubator at 37 °C. At 4.5 d.p.c., RNA was extracted using ReliaPrep RNA MiniPrep System (Promega, WI, USA) and analyzed using SAGE-seq (Sequence Analysis of Gene Expression) (GSE268174; GSM8287256–GSM8287273). Differentially expressed genes (DEGs) were identified using RaNA-seq and iDEP (version: 1.1) [13, 14] with criteria of FDR (False Discovery Rate) < 0.1 and fold change = 2.

2-cell stage embryos or blastocysts were transplanted into oviducts or uteri of the pseudo-pregnant ICR mice (Jackson Laboratory Japan, Yokohama, Japan or Japan SLC), respectively. About 10 embryos were transferred per oviduct or uterine horn. Anesthesia followed a mixture of three anesthetics and atipamezole [15]. Mice were kept warm on a 35 °C heating pad (KN-475–3-35, Natsume Seisakusho, Tokyo, Japan) until awake, with carprofen for pain management. Some mice were euthanized four days post-transplantation for implantation rate calculation; others were kept for breeding and fertility checks through sibling mating.

### Questionnaire survey

An anonymous questionnaire survey was conducted at Yamaguchi University; Quantitative analysis was performed by scoring based on the ranking of responses.

### Ethics

Animal experiments were approved by the Yamaguchi University Animal Use and Care Committee (Approval Number: 414) and the Laboratory Animal Ethics Committee of ARK Resource (Approval Number: AW-23059), and subsequently conducted at ARCLAS (Advanced Research Center for Laboratory Animal Science) during the AAALAC International Accreditation period (2018–2023) and at the Central Laboratory of ARK Resource, respectively. The human participant survey was approved by the Yamaguchi University Review Committee for Non-Medical Research Involving Human Participants (Approval Number: 2022–087-01).

### Statistical analysis

The rates were evaluated using a binomial test, while values were assessed using Student’s *t*-test. Statistical significance was defined as *p* < 0.05. For multiple comparison, Bonferroni correction was applied. Statistical significances are indicated by an asterisk (*) in the figures and tables.

## Results

### Coloration and cytotoxicity of dye-added media

We dissolved 32 substances, mainly food additive dyes, in DPBS or DMSO to determine concentrations with significant color variations (Fig. S1).

**Fig. 1:**
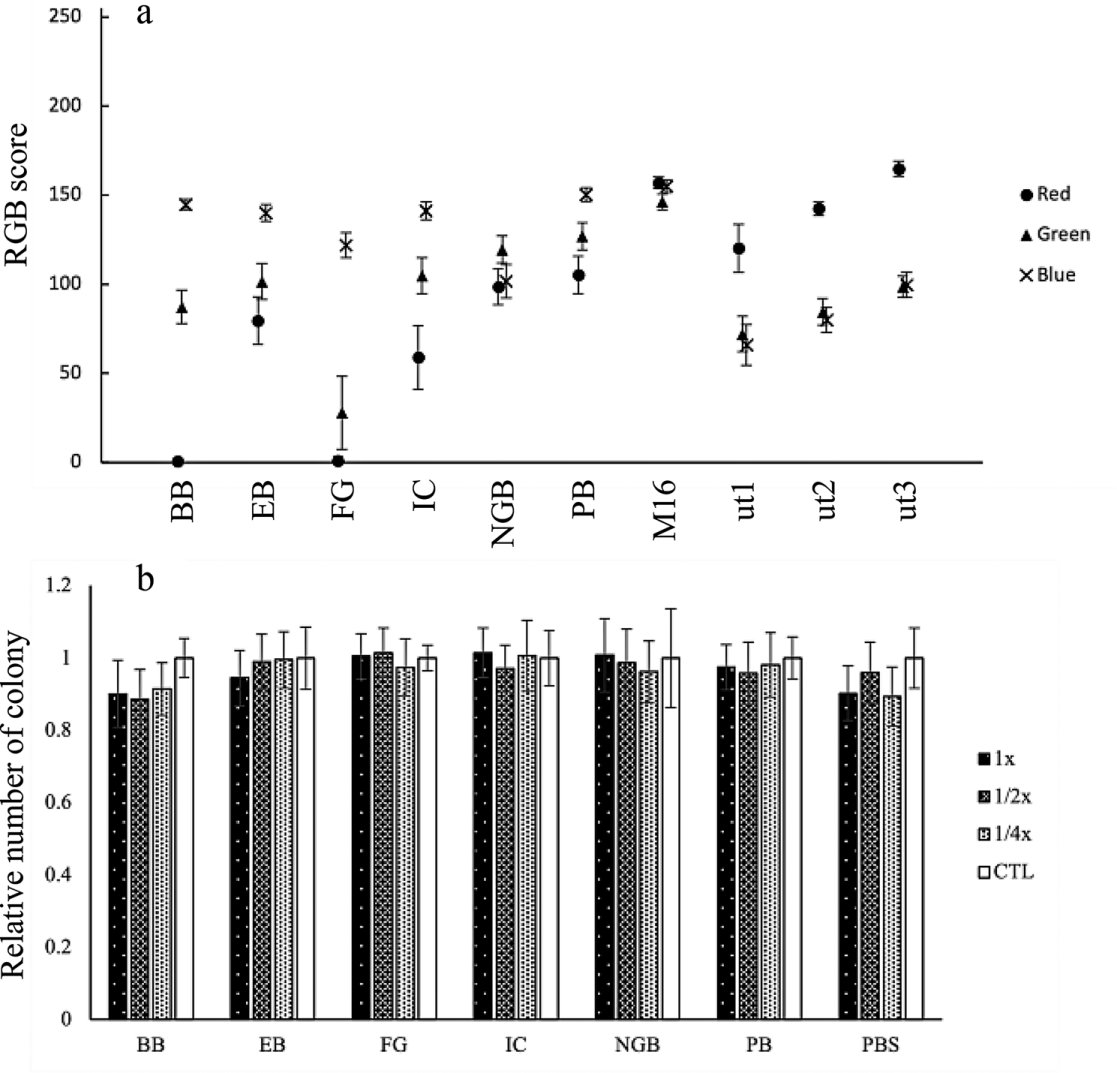
Properties of the dye-added culture medium. **a**) The color tone of the selected dye-added culture medium is shown. M16 (non-additive) and mouse uteri (ut1-3) were used as controls. **b**) The selected results of the colony formation inhibition assay of the dye-added culture medium are shown. The relative number of colonies in the control was set to 1. Error bars represent ± standard error of mean. Data for other dyes are shown in Fig. S2

We set the concentration where the dye did not fully dissolve and maintained a strong color tone from photographs and established this concentration as the final concentration (Table 1). The concentration was determined by comparing the dye color with that of the M16 medium, identifying the lowest concentration at which a noticeable color difference was observed even upon two-fold dilution, as consistently judged by multiple experimenters. Compared to the non-additive control; M16, the R (red) and G (green) values were significantly decrease especially in the Brilliant Blue FCF (BB) and Fast Green FCF (FG) (Fig. 1a), with the R-value being lower than the uterine (ut1-3) R-value. Colony formation inhibition assays showed that seven substances (Acid Black I (ABI), Brilliant Cresyl Blue (BCB), Janus Green (JG), Methylene Blue (MB), Methyl Green (MG), Nile Blue (NB), and Quinizarin Blue (QB)) had high cytotoxicity, preventing colony formation even at 1/4× concentration (Fig. S2).

Twelve compounds (Alizarin Cyanin Green F (ACGF), Bromophenol Blue (BPB), Chicago Sky Blue (CSB), Eumelanin (EM), Gardenia Blue (GB), Indocyanine Green (ICG), Light Green SY Yellowish (LGY), Phloxine B (Ph), Rose Bengal (RB), Sodium Copper-chlorophyllin (SCC), Trypan Blue (TB), and Quinizarin Green SS (QG)) displayed concentration-dependent cytotoxicity (Fig. S2). Eleven substances (BB, Evans Blue (EB), FG, Indigo Carmine (IC), Naphthol Green B (NGB), Patent Blue (PB), Acid Green 3 (AG3), Acid Red 52 (AR), New coccine (NC), Sunset Yellow FCF (SY), and Tartrazine (T)) showed limited cytotoxicity, similar to the non-additive control (Fig. 1b, Fig. S2). AG3 was excluded due to color tone attenuation, and AR, NC, SY, and T were excluded due to insufficient red value decrease. Comet assays confirmed that the remaining six dyes showed no comets at 1x concentrations (Fig. S3).

Thus, we identified six dyes with low cytotoxicity and a shift toward the blue spectrum.

### Effect of dye addition to the medium on embryogenesis

We analyzed the effects of six identified dyes on embryonic development, First, we compared the developmental rates up to the blastocyst stage. Differences were observed in the developmental rate when IC was used compared to the control M16; however, no significant difference was detected (Table 2; statistical significance was set at *p* < 0.05/6 = 0.0083 after Bonferroni correction). Subsequent analyses were conducted for the five dyes, excluding IC.

**Table 2:** Developmental rates to blastocysts in the dye-added medium

	Total number of Embryos	Number of blastocysts	Percentage of blastocysts (%)	*P* value
FG(1x)	128	122	95	0.153
EB(1x)	223	220	99	0.015
BB(1x)	123	119	97	0.181
PB(1x)	119	117	98	0.095
NGB(1x)	120	114	95	0.143
IC(1x)	129	119	92	0.020
CTL(M16)	124	119	96	-
CTL(KSOM)	157	154	98	(0.075)
CTL(mWM)	155	152	98	(0.079)

**Fig. 2:**
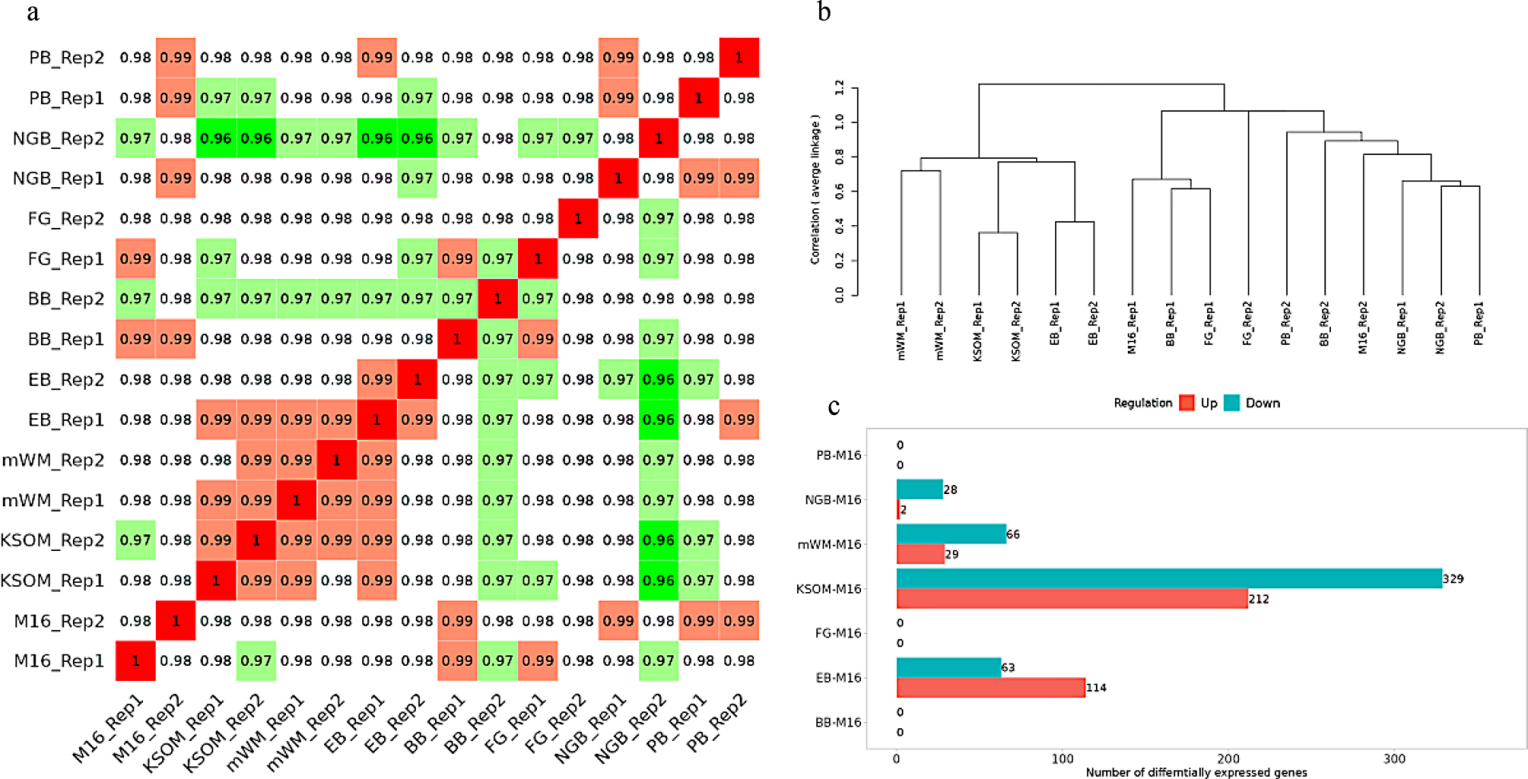
Global gene expression analysis of blastocysts after culturing in dye-added culture medium. **a**) Correlation heat map of global gene expression in blastocysts after culturing in each medium. The color scale represents the Pearson correlation coefficient, with green indicating lower correlation and red indicating higher correlation. **b**) Hierarchical clustering tree of the top 75% expressing genes. **c**)Numbers of differentially expressed genes for each medium compared to the control medium

**Fig. 3:**
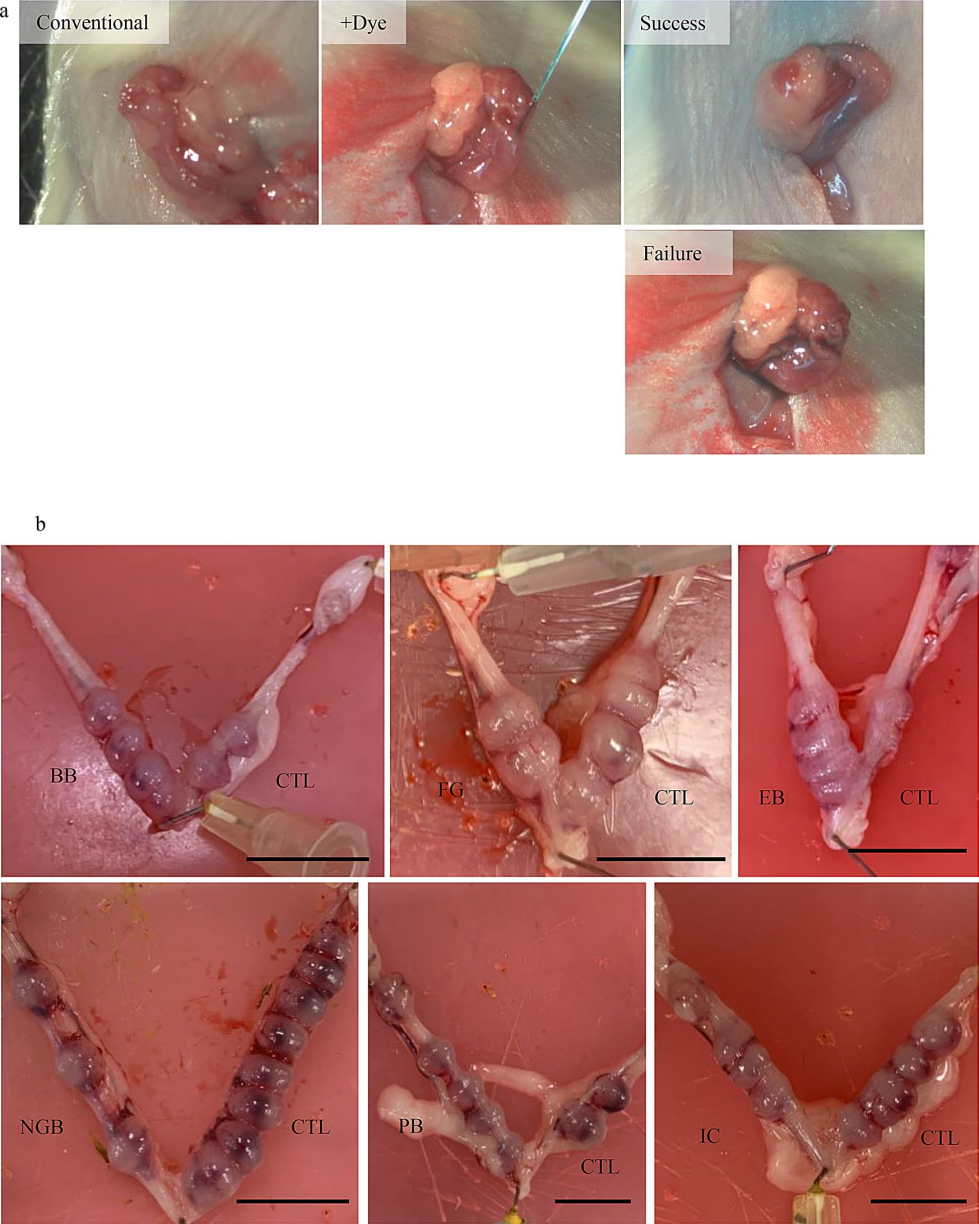
Embryo transfer experiment. **a**) Photographs of embryo transfer. From left to right: embryo transfer using the conventional method, embryo transfer using dye-added medium, and post-embryo transfer. The bottom row shows a failed transfer. **b**) The E8.5 uterus after embryo transfer. Experimental conditions are shown near the uterine horn. Bars, 1.0 cm

Next, we cultured embryos up to the blastocyst stage using these culture media and performed a comprehensive gene expression analysis. Correlation analysis showed gene expression correlations of 0.96 or higher between any pairs of groups (Fig. 2a). Cluster analysis revealed that embryos cultured in EB-added medium were classified into the mWM-KSOM cluster, whereas the other addition groups were classified within the M16 lineage (Fig. 2b). Furthermore, when we searched for DEGs using the M16 group as a reference, the KSOM group presented over 500 genes, whereas in these five addition groups, the detection frequency was lower (Fig. 2c). In particular, EB showed 177 DEGs, and NGB showed 30 DEGs, but no DEGs were detected in BB, FG, and PB. These results indicate that the impact of these dyes on embryonic development was very limited, and the impact of differences in culture media was smaller than expected. KEGG pathway enrichment analysis of the DEGs from each of groups did not reveal any significant enriched pathways. Next, we used the dye culture media as actual embryo transfer solutions. Compared with conventional embryo transfer procedures, glass capillaries were easier to visualize with dye-added transfer solutions. Additionally, the success of embryo transfer could be determined by the distribution of the transfer solution inside and outside the uterus (Fig. 3a). Observation of uteri at 8.5 days post-transfer revealed implantation sites for all transfer solutions (Fig. 3b). The number of transfers and implantations are listed in Table 3. Although a significant difference was observed only in the BB groups (statistical significance was set at *p* < 0.05/7 = 0.0071 after Bonferroni correction), there was generally no significant decrease in implantation rates compared to the control.

**Table 3:** Summary of embryo transfer using dye-added medium

	Number of embryo transferred	Number of implantations	Implantation rate (%)	*P* value
FG	55	18	33	0.082
EB	62	30	48	0.025
BB	66	40	61	0.0001
BB#2	55	39	71	0.000001
NGB	35	12	34	0.127
PB	35	18	51	0.037
IC	50	19	38	0.116
CTL	173	66	38	0.062

BB#2: Experimenter#2

### Outcome after dye-added embryo transfer

We examined whether successful pregnancies could be achieved embryo transfer using dye-added media, confirming that the dyes did not inhibit implantation. Specifically, we used BB and FG as dye groups with minimal impact on embryos, and EB and PB as a group with potential impact, were tested. After embryo transfer, we waited until full-term pregnancy to determine whether offspring could be obtained. As a result, we successfully obtained offspring from all three dye-added media (Fig. S4a).

**Fig. 4:**
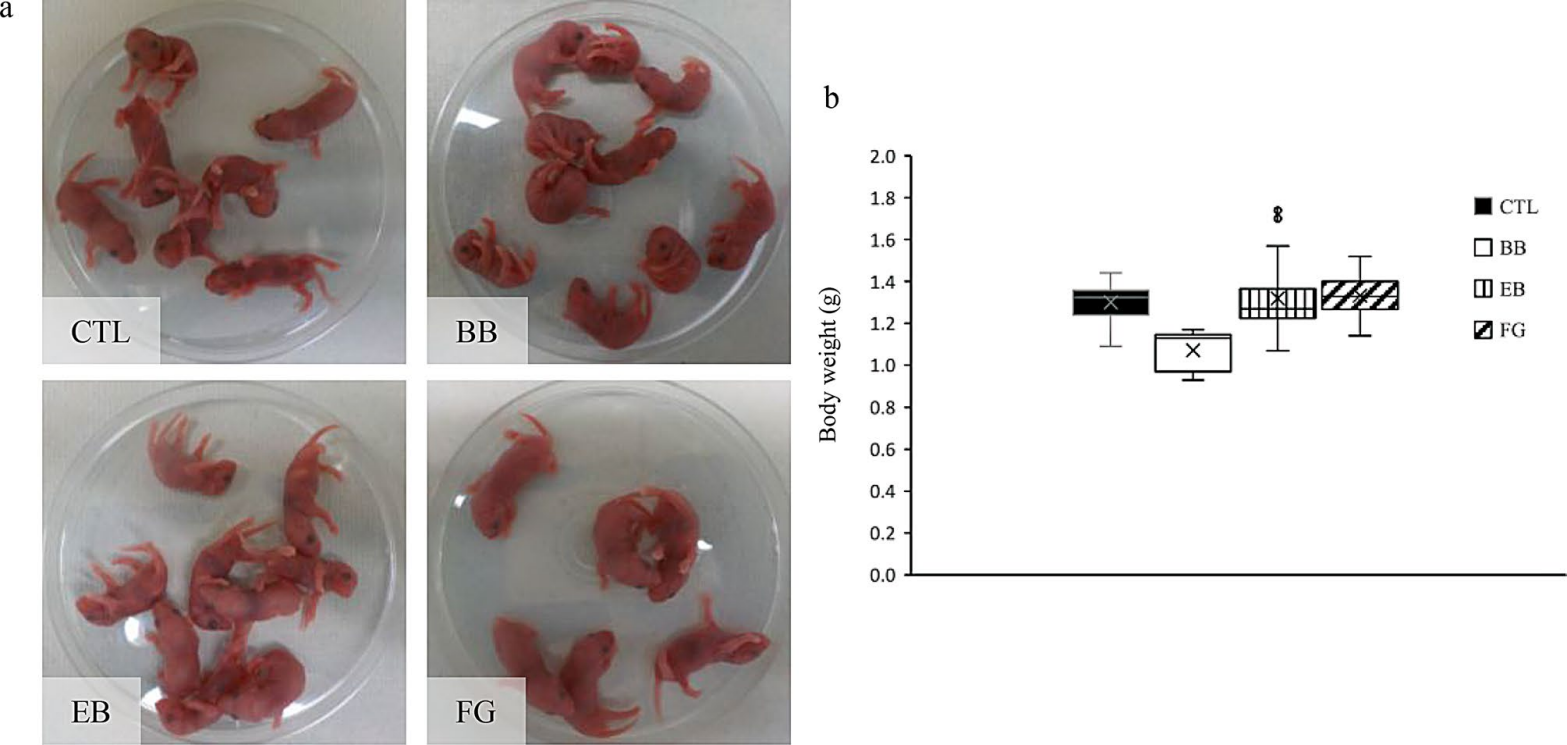
Offspring of embryo-transfer-derived mice. **a**) Neonates of the next generation of individuals obtained by embryo transfer. The conditions of each experiment are shown at the lower of the photo. **b**) Body weight of a neonate from the next generation obtained by embryo transfer. Error bars represent ± standard error of mean

We measured and observed the growth of the offspring, both males and females, and found no significant differences compared to the control group, except for the FG group females at four weeks (Fig. S4b, c, *p* = 0.013 < 0.05/3). The results of oviduct transplantation demonstrated an improvement in the number of offsprings obtained per transplant experiment (Table 4; *p* < 0.05) and a successful reduction in the probability of embryo transfer failure (the number of mice with pregnancy failure after embryo transfer; BB: 4/49, CTL: 10/49, *p* < 0.05). Subsequently, we conducted sibling mating to check fertility and successfully obtained the next generation from individuals in each group (Fig. 4a). The newborn weights were also comparable to those in the control group (Fig. 4b). Overall, the identification of dye additives for embryo transfer that does not affect fertility was successful in this experiment.

**Table 4:** Summary of embryo and offspring in oviduct transfer

	Number of embryo transferred	Number of offsprings	Delivary Rate (%)	*P* value
CTL	944	284	30.1	-
BB	893	329	36.8	2 × 10−6

### Subjective evaluation of dye-added medium

Finally, we evaluated the improvement in the visibility of the dyes identified in the embryo transfer media. We compared the visibility of glass capillaries filled with each transfer solution using a questionnaire survey. The questionnaire form is shown in Fig. S5.

**Fig. 5:**
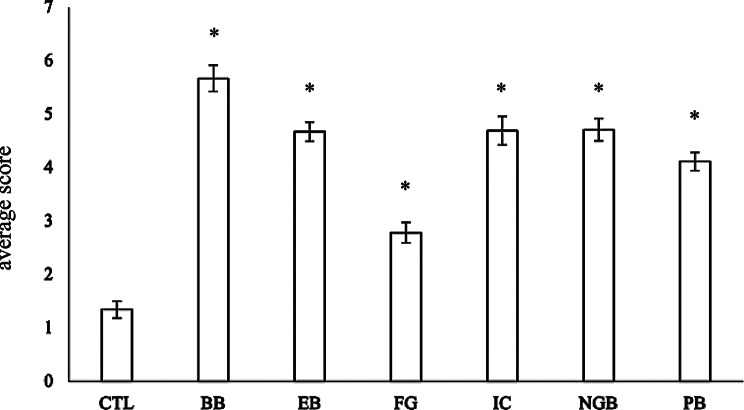
Quantification of visibility. The scoring results of the visibility questionnaire are shown below. The contents of the questionnaire are shown in Fig. S5. Bar ± standard error of mean. *: *p* < 0.05/6 ≈ 0.008

The results demonstrated that the visibility of the media with each added dye was improved compared to the control M16 (Fig. 5; *p* = 10^−25^ − 10^−8^).

## Discussion

In this study, we primarily utilized food-additive dyes to ensure high embryonic development rates while addressing the low visibility issue in conventional embryo transfer procedures. This approach not only enables verification of the completeness of embryo transfer but also enhances the productivity of both senior and novice researchers. Additionally, it contributes to a reduction in animal usage for training in embryo transfer techniques.

For dye selection, we verified candidates primarily based on those used as food and pharmaceutical additives in Japan as well as dyes commonly used in animal experimentation and cell biology. Variations in color tone were observed at concentrations of 0.02% or less for all dyes. The potential impact of Trypan Blue on fetal development has been discussed for some time [16, 17], although some reports have suggested that Trypan Blue does not affect the development of rabbit-cultured embryos [18]. Despite its common use in general culture, it should not be used for embryo transfer. Copper Chlorophyllin Sodium reduces cell proliferation at a concentration of 100 µM (approximately 0.007%) [19]. A 13 µM solution of Brilliant Cresyl Blue (approximately 5.2 × 10^−4^%) shows no toxicity to human follicular cells [20]. Additionally, low concentrations (26 µM; approximately 1 × 10^−3^%) and short exposure times of dyes like Brilliant Blue have been shown to allow for embryo selection without affecting embryonic development in animals such as cattle, rabbits, and cats [21–23]. Fast Green FCF, a food colorant, has been used to visualize viral liquids in chicken developmental genetic studies [24]. Brilliant Blue was recently reported as an alternative [25]. It can be inferred that food-grade dyes have minimal effects, even when exposed during embryonic development, as supported by the results of the present study. Evans Blue is widely used as a tracer for veins by binding to albumin [26], and has been shown to have minimal direct toxicity or effects on embryos. Evans Blue and Indigo Carmine can also be used to track rat fetuses in utero [27], suggesting low toxicity. Patent Blue and Indigo Carmine have been reported for clinical applications in the nervous system (0.1%) and in regeneration experiments (5% IV), indicating their high safety [28, 29]. However, little information is available on Naphthol Green B. Nonetheless, these six dyes did not show significant cytotoxicity within the concentration ranges tested in this study.

Among the six dyes tested in this study, none had a significant impact on blastocyst formation rate, and three dyes showed minimal effects on gene expression levels. One of these dyes, Fast Green, has already been used for visualization of ribonucleoprotein solution in in vivo genome editing [9], and this study confirmed its safety. In addition, Patent Blue and Brilliant Blue have been proposed as potential substitutes for Fast Green. Furthermore, no effects were observed on the implantation, subsequent embryonic development, or fertility of the next generation. Although changes in gene expression related to implantation and placental formation have been reported in embryos from natural mating and in vitro fertilization [30], this study showed almost negligible effects. The use of these dyes in embryo transfer solutions is considered safe. Although some dyes showed variations in gene expression upon addition, global expression differences were observed among the non-addition groups (M16, KSOM and mWM). Although similar phenomena have been reported when comparing KSOM and mWM [31], we did not observe similar trends in the expression of these genes (data not shown). Subtle differences in composition due to supplier variations, batch differences, or parental mouse breeding conditions may have an impact. Although all experiments were conducted under the same conditions in this study, it is believed that these dyes do not disrupt embryos at the gene expression level and have minimal effects on implantation, birth, and fertility in the next generation.

Finally, when comparing the visibility of the culture media with added dyes to those without dyes, improvements were observed for all six dyes tested, including the three promising candidates. In conclusion, this study identified six compounds (BB, EB, FG, IC, NGB, and PB) as additives in culture media that provide both safety and enhanced visibility.

## Conclusions

This study identified six dyes including BB, EB, FG, IC, NGB and PB that enhance the visibility of embryo transfer procedures without compromising embryonic development, implantation, or fertility (Table 5). Among them, Fast Green FCF and Brilliant Blue FCF at a concentration of 0.01% were particularly promising, showing minimal effects on gene expression. These dyes offer a safe and effective alternative for improving embryo transfer efficiency while reducing the need for additional animal use in training.

**Table 5:** Summary of screening results for nontoxic dyes in mouse embryo transfer

	color	cytotoxicity	embryo development	visibility
BB	Blue	→	↑	↑
EB	Blue	→	→	↑
FG	Blue	→	→	↑
IC	Blue	→	→	↑
NGB	Green	→	→	↑
PB	Blue	→	→	↑
*See Fig.*	Fig. 1, S1	Fig. 1, S2, S3	Figs. 2, 3, 4, S4	Fig. 5, S5
*See Table*	-	-	Tables 2, 3, 4	-

## Electronic supplementary material

Supplementary Material 1

## Data Availability

SAGE-seq results are published under accession number: GSE268174; GSM8287256-GSM8287273.

## Abbreviations

ABI: Acid Black I
ABM: Acid Brown M
ACGF: Alizarin Cyanin Green F
AG3: Acid Green 3
AR: Acid Red 52
BB: Brilliant Blue FCF
BBr: Bismarck Brown Y
BCB: Brilliant Cresyl Blue
BPB: Bromophenol Blue
CSB: Chicago Sly Blue 6B
EB: Evans Blue
EM: Eumelanin
FG: Fast Green FCF
GB: Gardenia Blue
IC: Indigo Carmine
ICG: Indocyanine Green
JG: Janus Green
LGY: Light Green SY Yellowish
MB: Methylene Blue
MG: Methyl Green
NB: Nile Blue A
NC: New Coccine
NGB: Naphthol Green B
PB: Patent Blue
Ph: Phloxine B
RB: Rose Bengal
SCC: Sodium Copper-chlorophylin
SY: Sunset Yellow FCF
T: Tartrazine
TB: Trypan Blue
QB: Quinizarin Blue
QG: Quinizarin Green SS
