# Supplementary material for: Screening of nontoxic dyes for improved visualization and success rate in mouse embryo transfer
Source: Lab Anim Res. 2025 Dec 1;41:30. doi: 10.1186/s42826-025-00261-7 (PMC12667083; doi:10.1186/s42826-025-00261-7)
Supplement: Supplementary file 1 — Supplementary Material 1 [file 42826_2025_261_MOESM1_ESM.pptx]

## Slide 1
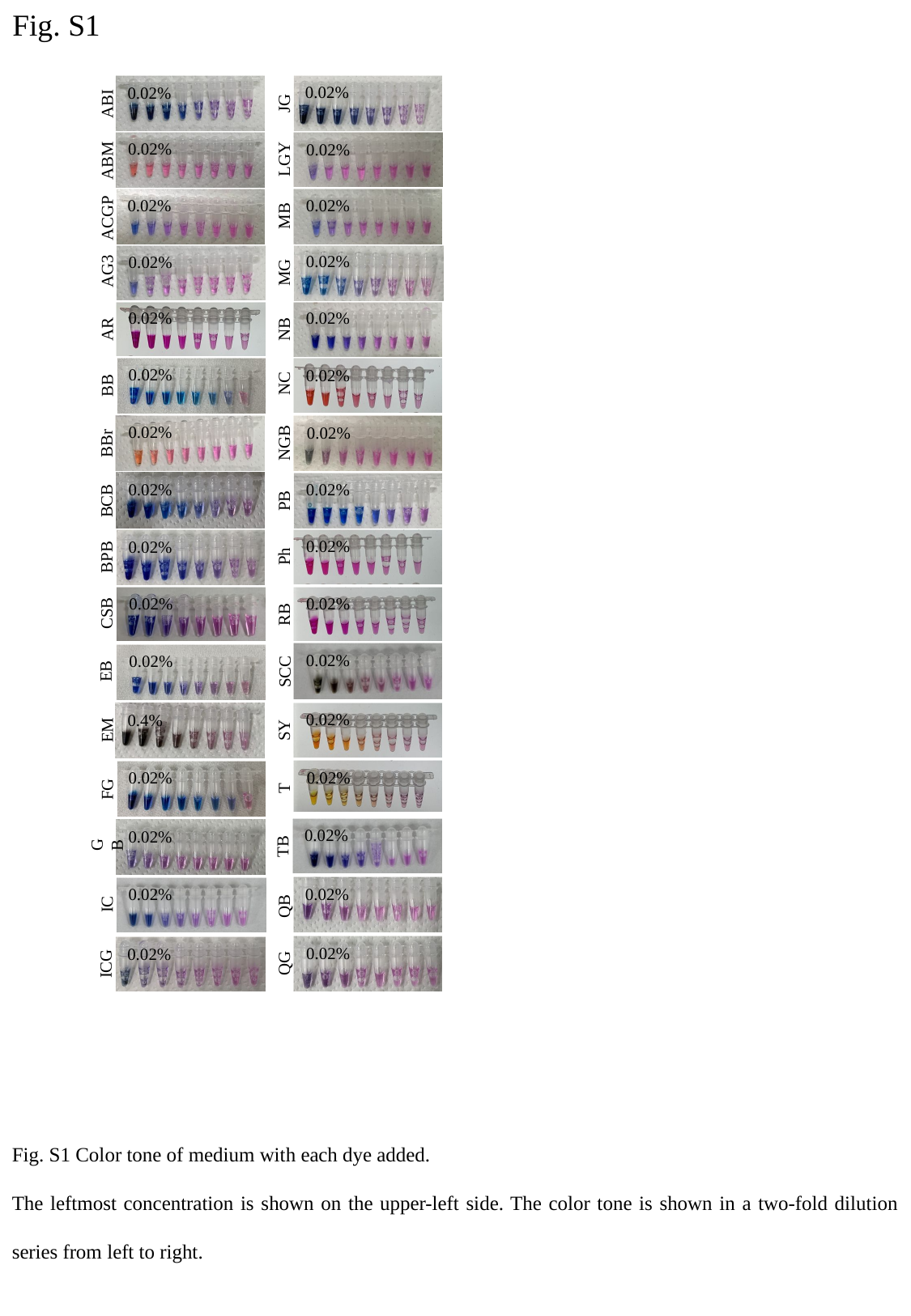

Fig. S1
0.02%
0.02%
JG
ABI
0.02%
0.02%
LGY
ABM
0.02%
0.02%
MB
ACGP
0.02%
0.02%
AG3
MG
0.02%
0.02%
AR
NB
0.02%
0.02%
NC
BB
0.02%
0.02%
NGB
BBr
0.02%
0.02%
PB
BCB
0.02%
0.02%
Ph
BPB
0.02%
0.02%
CSB
RB
0.02%
0.02%
EB
SCC
0.02%
0.4%
EM
SY
0.02%
0.02%
T
FG
0.02%
GB
0.02%
TB
0.02%
0.02%
IC
QB
0.02%
0.02%
QG
ICG
Fig. S1 Color tone of medium with each dye added.
The leftmost concentration is shown on the upper-left side. The color tone is shown in a two-fold dilution series from left to right.

## Slide 2
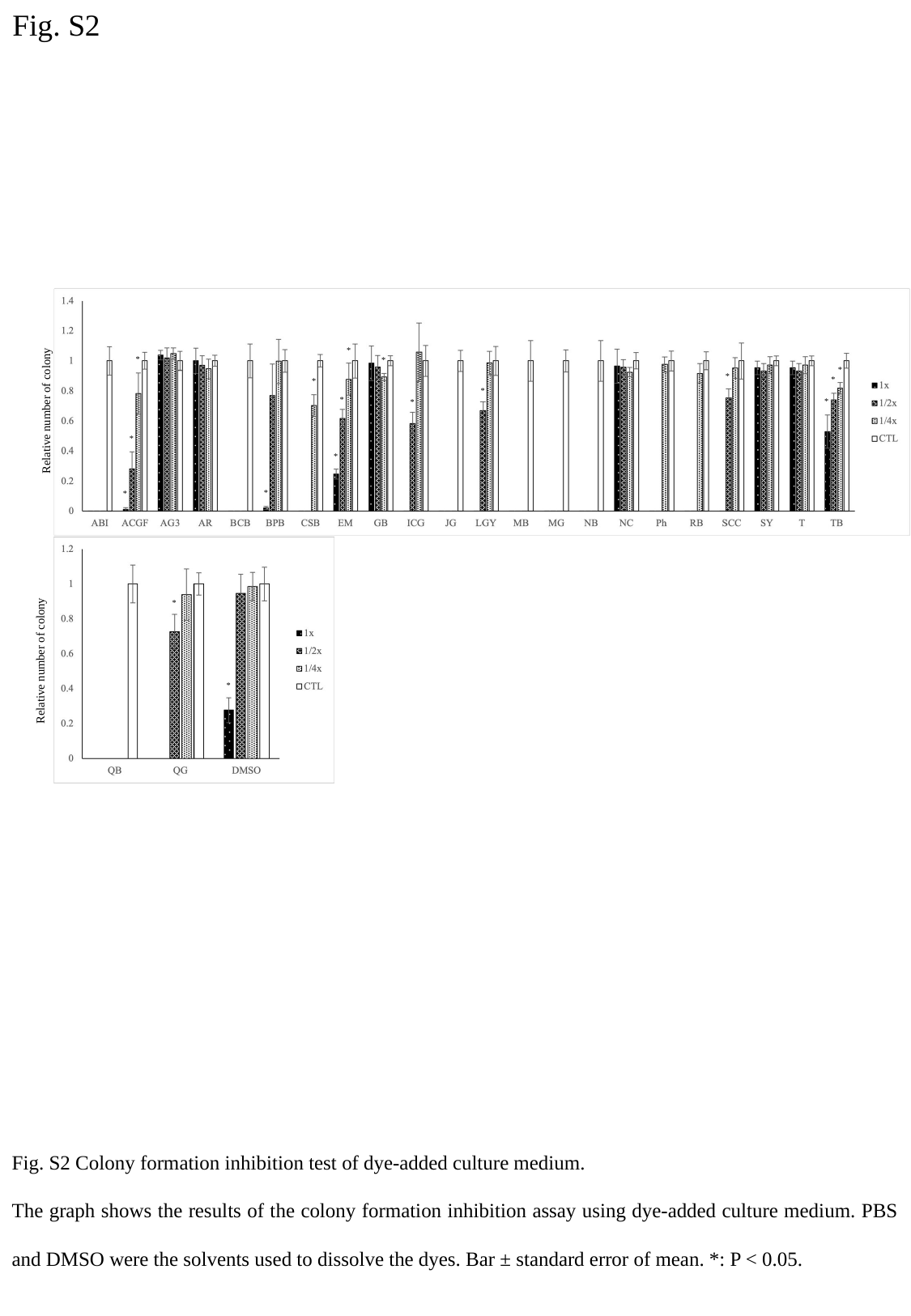

Fig. S2
*
*
*
*
*
*
*
*
*
*
*
*
*
*
*
Relative number of colony
*
*
Relative number of colony
Fig. S2 Colony formation inhibition test of dye-added culture medium.
The graph shows the results of the colony formation inhibition assay using dye-added culture medium. PBS and DMSO were the solvents used to dissolve the dyes. Bar ± standard error of mean. *: P < 0.05.

## Slide 3
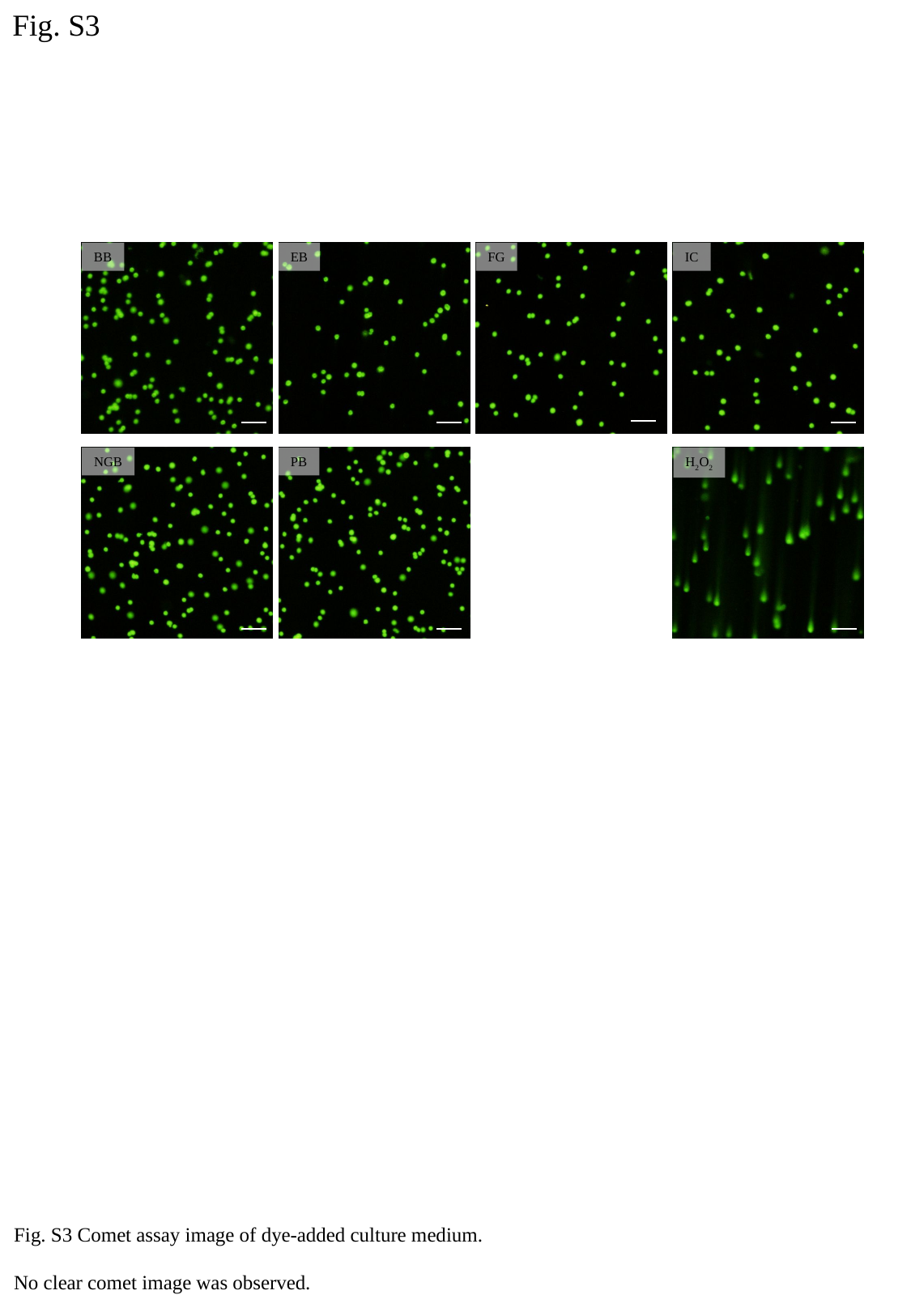

Fig. S3
BB
EB
FG
IC
NGB
PB
H2O2
Fig. S3 Comet assay image of dye-added culture medium.
No clear comet image was observed.

## Slide 4
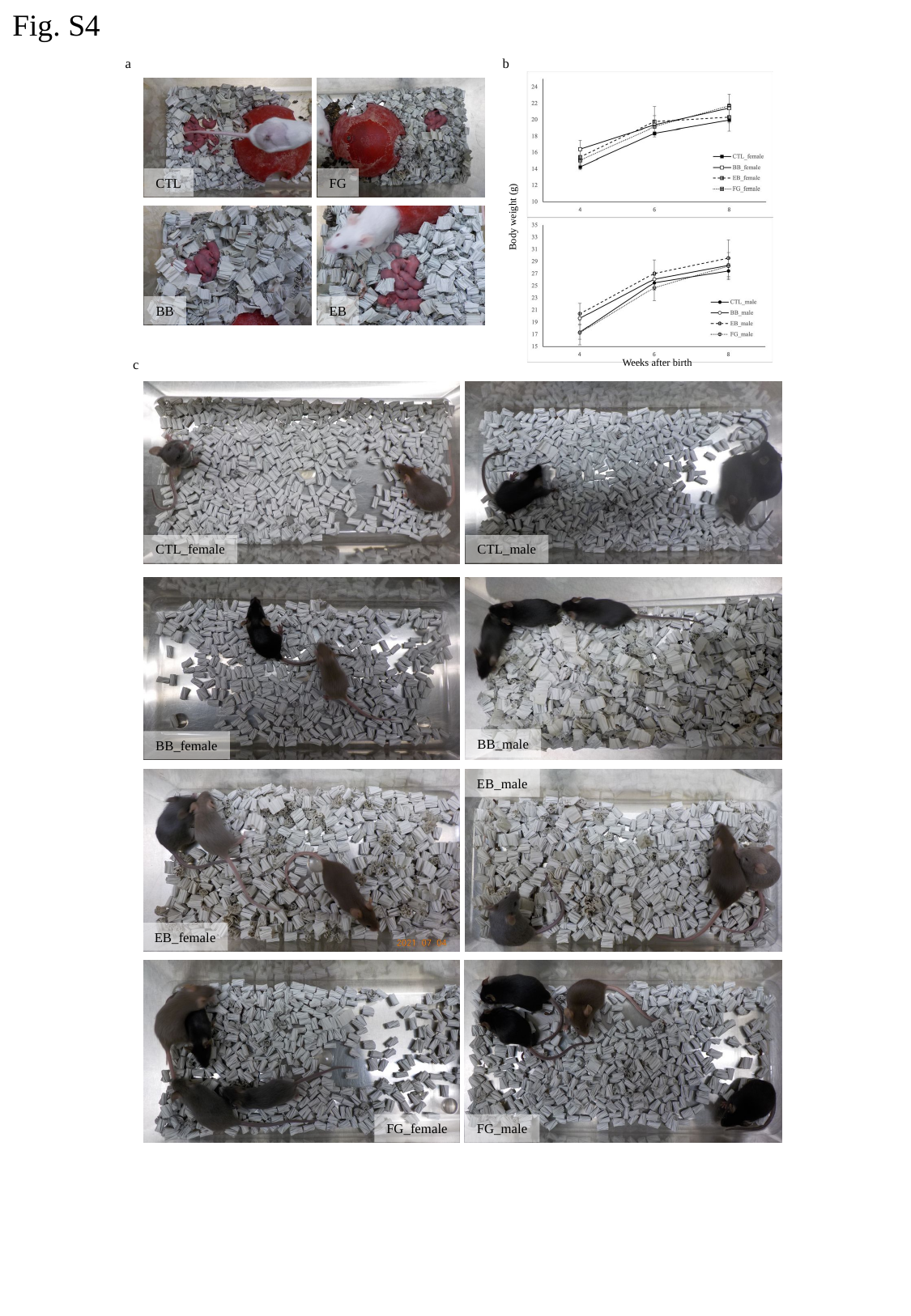

Fig. S4
a
b
Body weight (g)
Weeks after birth
FG
CTL
BB
EB
c
CTL_female
CTL_male
BB_male
BB_female
EB_male
EB_female
FG_female
FG_male

## Slide 5
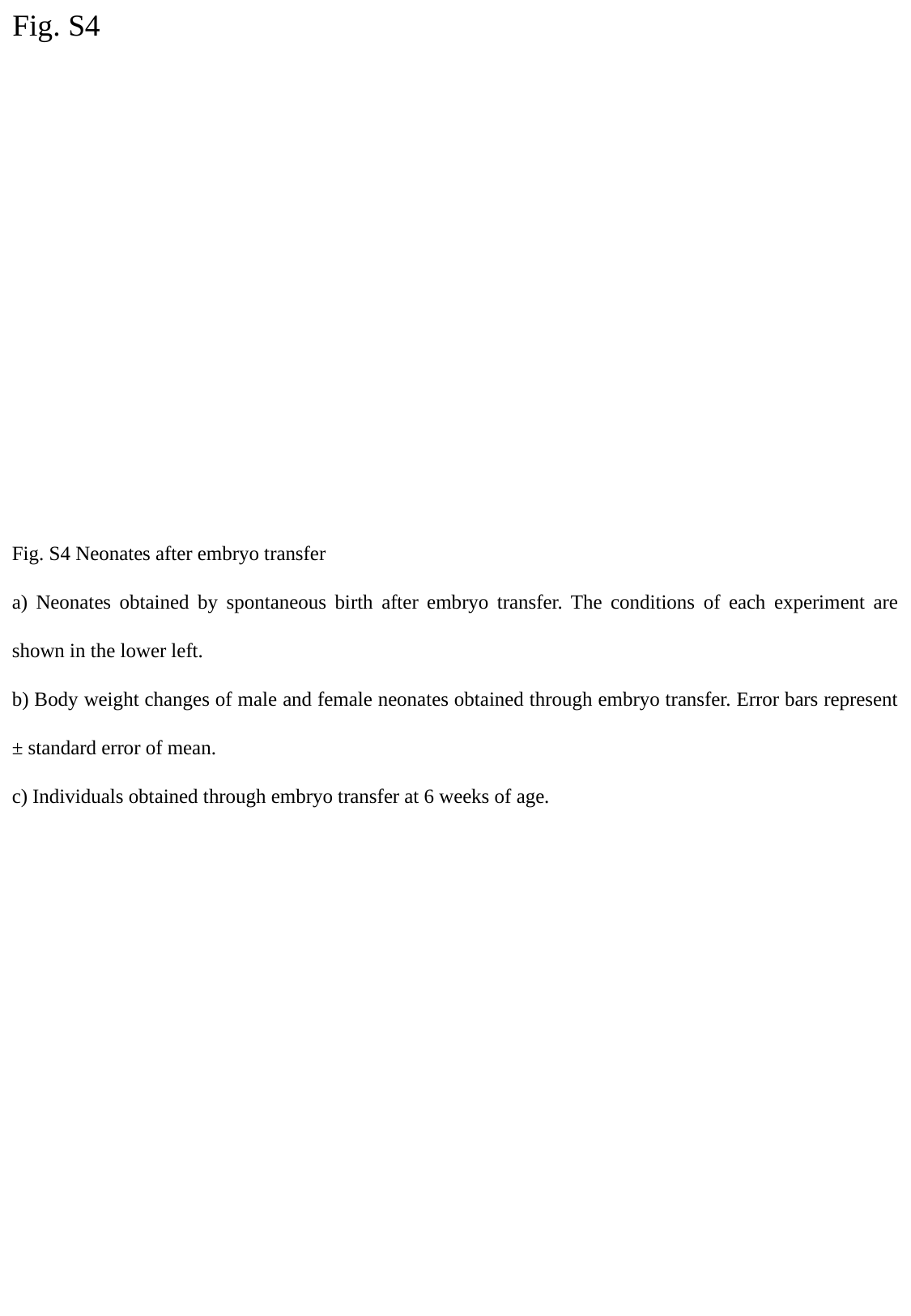

Fig. S4
Fig. S4 Neonates after embryo transfer
a) Neonates obtained by spontaneous birth after embryo transfer. The conditions of each experiment are shown in the lower left.
b) Body weight changes of male and female neonates obtained through embryo transfer. Error bars represent ± standard error of mean.
c) Individuals obtained through embryo transfer at 6 weeks of age.

## Slide 6
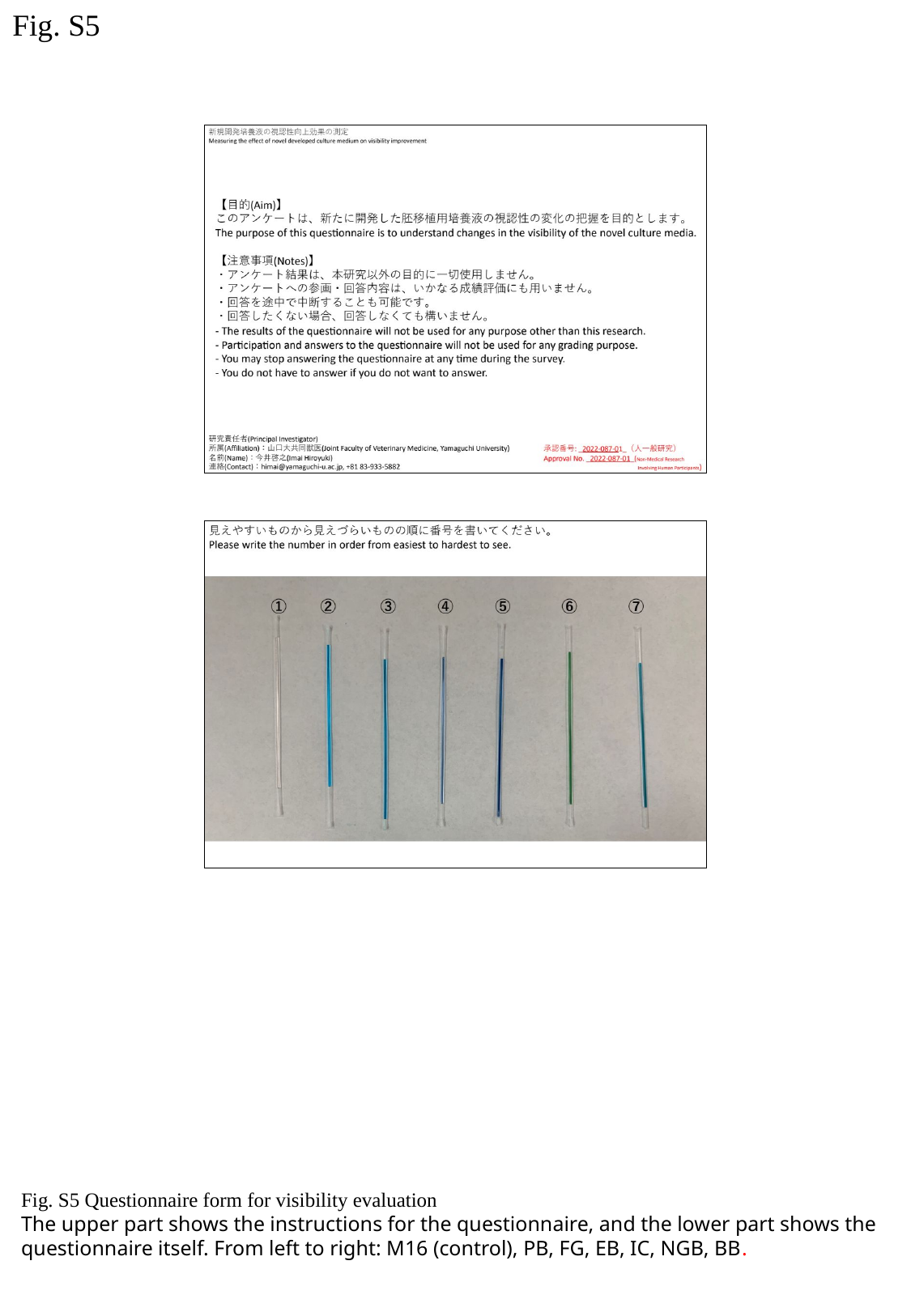

Fig. S5
Fig. S5 Questionnaire form for visibility evaluation
The upper part shows the instructions for the questionnaire, and the lower part shows the questionnaire itself. From left to right: M16 (control), PB, FG, EB, IC, NGB, BB.
